# Emergent communication of multimodal deep generative models based on Metropolis-Hastings naming game

**DOI:** 10.3389/frobt.2023.1290604

**Published:** 2024-01-31

**Authors:** Nguyen Le Hoang, Tadahiro Taniguchi, Yoshinobu Hagiwara, Akira Taniguchi

**Affiliations:** ^1^ Graduate School of Information Science and Engineering, Ritsumeikan University, Kusatsu, Shiga, Japan; ^2^ College of Information Science and Engineering, Ritsumeikan University, Kusatsu, Shiga, Japan; ^3^ Research Organization of Science and Technology, Ritsumeikan University, Kusatsu, Shiga, Japan

**Keywords:** symbol emergence, emergent communication, multimodal, deep generative model, variational autoencoder, Metropolis-Hastings, naming game

## Abstract

Deep generative models (DGM) are increasingly employed in emergent communication systems. However, their application in multimodal data contexts is limited. This study proposes a novel model that combines multimodal DGM with the Metropolis-Hastings (MH) naming game, enabling two agents to focus jointly on a shared subject and develop common vocabularies. The model proves that it can handle multimodal data, even in cases of missing modalities. Integrating the MH naming game with multimodal variational autoencoders (VAE) allows agents to form perceptual categories and exchange signs within multimodal contexts. Moreover, fine-tuning the weight ratio to favor a modality that the model could learn and categorize more readily improved communication. Our evaluation of three multimodal approaches - mixture-of-experts (MoE), product-of-experts (PoE), and mixture-of-product-of-experts (MoPoE)–suggests an impact on the creation of latent spaces, the internal representations of agents. Our results from experiments with the MNIST + SVHN and Multimodal165 datasets indicate that combining the Gaussian mixture model (GMM), PoE multimodal VAE, and MH naming game substantially improved information sharing, knowledge formation, and data reconstruction.

## 1 Introduction

Emergent communication (*EmCom*) is vital in developing computational models that allow artificial agents to use sign systems and form internal representations of their environments (Cangelosi and Parisi, 2002; Steels, 2003). Rooted in the principles of human language and communication, *EmCom* particularly focuses on how the process of semiosis leads to the emergence of signs. Semiotics, as theorized by Peirce (Peirce, 1991), view the sign not as a static entity but as something that emerges and evolves through the process of semiosis. In symbol emergence systems, this concept is replicated in artificial agents, enabling them to develop their own communication systems. These systems are based on semiotic principles, where signs (including symbols) emerge and gain meaning in the context of agent interactions and their environment (Taniguchi et al., 2016). The growth of this research area in artificial intelligence is underscored by efforts to construct multi-agent systems capable of understanding human language and cognition (Lazaridou et al., 2017).

Several models, such as the referential signaling game (Lewis, 2008) and naming game (Steels and Loetzsch, 2012), have explored *EmCom*, utilizing feedback mechanisms to refine coordination and vocabulary. In contrast, a recent approach called the Metropolis-Hastings (MH) naming game offers a different approach to *EmCom* which does not rely on explicity feedback, but rather on a principle of joint attention where both agents focus on the same observation (Hagiwara et al., 2019). This principle is hypothesized to be critical in the developmental stages of human infants around nine to 15 months and is theorized to facilitate significant advancements in lexical acquisition and language development (Tomasello and Farrar, 1986; Carpenter et al., 1998). The MH naming game employs a unique probability-based approach to evaluate the acceptance and rejection of information during agent interactions based on the judgment ratio calculated using the MH algorithm (Hastings, 1970). By focusing on joint attention and incorporating acceptance probabilities, the agents can improve their abilities to exchange information and form shared signs or vocabularies (Taniguchi et al., 2023).

Simultaneously, the evolution of deep learning and neural networks has allowed researchers to expand the boundaries of *EmCom* systems (Lazaridou and Baroni, 2020). This development is notably relevant in the context of multimodal deep learning, which combines different data modalities to improve the modeling of diverse data (Suzuki and Matsuo, 2022). In *EmCom* systems, incorporating multimodal information can enrich learning by providing multiple viewpoints on a dataset for a more accurate and robust communication strategy (Taniguchi et al., 2019). By leveraging information from multiple sources, such as visual, auditory, and textual data, multimodal DGM can capture and exploit the complementary nature of different data types (Baltrušaitis et al., 2019).

Studies on *EmCom* have employed multimodal data, such as the Inter-MDM model, which utilizes a multimodal Dirichlet mixture model to combine modalities within a single agent framework (Hagiwara et al., 2022). Although promising, its absence of deep generative modeling limits its capacity to learn extracted features and reconstruct objects corresponding to signs. Meanwhile, the Inter-GMM + VAE model, based on joint attention principles, incorporates DGM for shared vocabulary development but falls short in handling multimodal objects (Taniguchi et al., 2023). In this study, we propose using a multimodal DGM for each agent in our *EmCom* systems to overcome these shortcomings.

Furthermore, this study examined the significance of modality weighting within agent-based *EmCom*. Although previous research has highlighted the role of weighting in multimodal contexts (Baltrušaitis et al., 2019; Sutter et al., 2021), a thorough examination of agent communication is yet to be conducted. By assigning a higher ‘weight’ or importance to a modality that an agent can more readily learn and categorize, we mirror behaviors observed in human communication, where emphasizing concepts that are easier to understand improves human comprehension. Hence, focusing on a more readily comprehensible modality can enhance the creation of shared signs or vocabularies between agents in multimodal settings.

**FIGURE 1:**
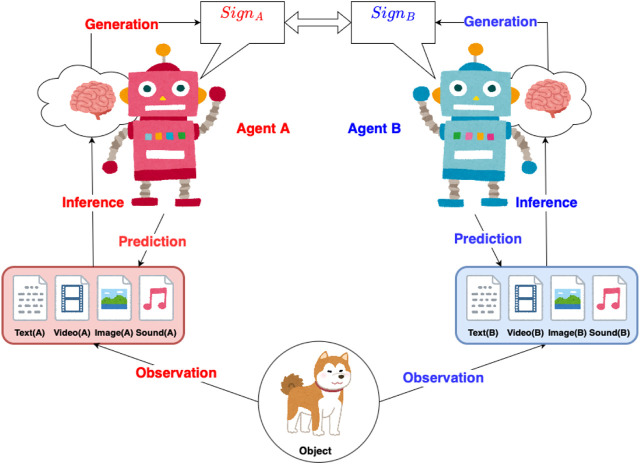
The *EmCom* between two multimodal agents: agents A and B observe a shared object, gathering multimodal data (text, video, image, sound, etc.). After inferring from this data, they generate and exchange signs. Using the received sign, each agent predicts the related multimodal data.

In the context of symbol emergence systems that employ DGM with multimodal data, three critical questions emerge that are yet to be addressed in previous works (Hagiwara et al., 2019; 2022; Taniguchi et al., 2023):

(1) Can we improve the categorization of multimodal data to facilitate symbol emergence by incorporating a multimodal DGM (as depicted in Figure 1)?
(2) Can the proposed model sustain the functionality of categorizing each agent through semiotic communication, even in scenarios with missing modalities?
(3) Given that different modalities may vary in their interpretability to agents, how does manipulating the emphasis or importance of these modalities affect agents’ ability to develop a shared understanding and interpretation of signs?.

Building upon Inter-GMM + VAE and employing variational autoencoders (VAE) (Kingma and Welling, 2013), our study aims to demonstrate that integrating an MH naming game with a multimodal VAE can advance the field of *EmCom*. In this study, we employed three widely used multimodal approaches within the structure of the multimodal VAE: product-of-experts (PoE) (Wu and Goodman, 2018), mixture-of-experts (MoE) (Shi et al., 2019), and mixture-of-product-of-experts (MoPoE) (Sutter et al., 2021). These approaches are crucial in determining how the VAE processes and integrates information from different modalities. The primary objective is to combine diverse multimodal information into a single comprehensive representation within a VAE (Suzuki and Matsuo, 2022). The main contributions of this study are as follows:

• We introduce *EmCom* models that employ multimodal VAE as agents, using MoE, PoE, and MoPoE in conjunction with the MH naming game. These address the challenge of extending the Inter-GMM + VAE to handle observations as multimodal data.
• We refine these models by incorporating the weight of each modality in multimodal VAE and adjusting the value of *β* to disentangle the latent space. We evaluate the impact of weight and *β* on the results. This contribution addresses the challenge of optimizing multimodal VAE by focusing on a modality the model can more easily learn and categorize.

Because of the model architecture, differing objectives, and multimodal nature of our models, a direct comparison with other methods is not feasible. Instead, we assessed the performances of these models in *EmCom* on various datasets and conditions. The experiments were conducted on two datasets: the benchmark dataset, MNIST + SVHN, which provides a controlled environment for evaluating our model’s performance, and the real-life dataset, Multimodal165, which examines the model’s ability to generalize and adapt to more diverse data. The experiments on the real-life dataset uncovered a limitation in the ability of the current model to represent real-life objects accurately. To address this issue, we employed hyperparameter tuning techniques to optimize the model parameters for more accurate representations of real-life objects. The remainder of this paper is structured as follows: an overview of related works (section 2), an introduction to the necessary preliminaries (section 3), a detailed description of our proposed model (section 4), experimental results on benchmark (section 5) and real-life datasets (section 6), and concludes with a discussion of our findings and suggestions for future research (section 7).

## 2 Related works

Emergent Communication (*EmCom*) studies focus on the evolution of communication systems among interactive agents, drawing from linguistics (Hurford, 2014) and human science (Linell, 2009). Research covers language emergence in human-human scenario (Okumura et al., 2023), and multi-agent systems, with studies examining population heterogeneity (Rita et al., 2022a), messaging efficiency (Lian et al., 2021), grammatical structures (Manning et al., 2020), language and agent co-evolution (Dagan et al., 2021), and the development of hierarchical reference systems (Ohmer et al., 2022).

Agents aim to perceive, create, and manipulate symbols to build a shared vocabulary through interaction and mutual adaptation (Wagner et al., 2003), leading to an emergent symbolic language grounded in their collective experiences (Steels, 2001). Key frameworks in this area include the referential signaling game (Lewis, 2008) and the naming game (Steels and Loetzsch, 2012), which aid in the development of coordination strategies and shared vocabularies through iterative feedback. The research in naming game, such as the creation of shared vocabulary (Baronchelli et al., 2006), the convergence of naming game (Vylder and Tuyls, 2008), also contribute to the topic.

Recent progress in deep learning has accelerated advances in *EmCom* systems, as evident in computer vision (Krizhevsky et al., 2012), natural language processing (Vaswani et al., 2017), and tasks that combine vision and language (Anderson et al., 2018; Zhou et al., 2020). These systems utilize deep generative models to address challenges such as developing efficient color-naming systems (Chaabouni et al., 2021), learning language structures (Gupta et al., 2021). Deep learning models have also advanced the study of compositionality and generalization in *EmCom*, fostering multi-agent cooperation with the emergence of language (Lazaridou et al., 2020), and allowing systems to form more complex messages (Chaabouni et al., 2020; Rita et al., 2022b; Xu et al., 2022). These contributions are thoroughly reviewed in (Galke et al., 2022; Brandizzi, 2023), which offer an extensive overview of the strides made in this domain.

Multimodal deep generative models have garnered considerable interest (Baltrušaitis et al., 2019). This led to a deeper understanding of underlying patterns and structures, resulting in communication systems with greater capabilities (Liang et al., 2023). These models have shown promise in learning joint representations across audio, video, and text (Ngiam et al., 2011), in applying graph structures to focus on relevant multimodal data (Veličković et al., 2018), and in generating image captions through a unified embedding space (Kiros et al., 2014). Additionally, multimodal learning with VAE has been explored using strategies such as JMVAE (Suzuki et al., 2016), TELBO (Vedantam et al., 2017), M2VAE (Korthals et al., 2019), and DMVAE (Lee and Pavlovic, 2020), PoE-MVAE (Wu and Goodman, 2018), MoE-MVAE (Shi et al., 2019), and MoPoE-MVAE (Sutter et al., 2021) to combine latent spaces. In the specific context of *EmCom*, multimodal data has been instrumental. Studies include exploring human-human interaction in multimodal discourse for emergent meaning-making (Krysanova, 2022) and multi-modal multi-step referential games to study agent communication (Evtimova et al., 2018). However, this approach typically processes different modalities independently for each agent rather than integrating them within a single agent.

## 3 Preliminaries

This section explores the core concepts that underpin our research: Metropolis-Hastings (MH) naming game, Variational Autoencoder (VAE) and multimodal VAE (MVAE).

### 3.1 Metropolis-Hastings naming game

Introduced in (Taniguchi et al., 2023), the MH naming game is a language game played by two agents. Typically, one agent observes an object and names it based on its perception drawn from its observations. This agent, playing as the speaker, communicates a word (i.e., a sign) by choosing from a posterior word distribution related to the object. The second agent, or the listener, decides whether to accept the sign based on its own understanding. The roles then switch between them. Notably, there is no direct feedback from the listener to the initial speaker. Direct feedback would involve the listener providing explicit responses or corrections to the speaker, thereby guiding the speaker’s future naming decisions. In contrast, the MH naming game features the joint attention, where both agents are aware of and focused on the same object. This shared focus ensures that the listener understands the context of the word or sign being used by the speaker, even though it does not offer direct corrective feedback.

*EmCom* systems are characterized by the inability of agents to see each other’s internal states, similar to how humans cannot view another’s thoughts (Steels, 2015). With its probability-based approach, the MH naming game enables agents to make inferences about the internal states of their counterparts based on the observed outcomes of their interactions. The MH naming game can be derived as an approximate Bayesian inference procedure for a specific probabilistic graphical model that represents two agents as an integrated system (Taniguchi et al., 2023).

**FIGURE 2:**
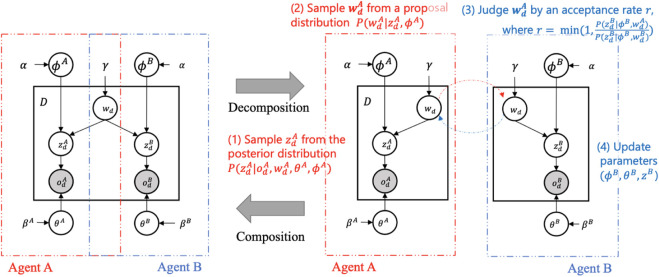
The MH naming game between two probabilistic generative models: When Agent A is a speaker, (1) it samples perceptual state $z_{d}^{A}$ from the observation $o_{d}^{A}$ of the *d*th object, then (2) samples a sign $w_{d}^{A}$ from $P\left(w_{d}|z_{d}^{A},\phi^{A}\right)$ and sends this sign to agent (B). Agent B, as a listener, (3) evaluates whether to accept this sign based on the acceptance rate *r*, then (4) updates its own parameters through a sampling process. Next, two agents swap their roles: Agent B speaks, and Agent A listens (Taniguchi et al., 2023)

Figure 2 illustrates the MH naming game between two probabilistic generative models involving a sequence of interactions between two agents, Agent A and Agent B. The graphical model in Figure 2 can be broken down into components corresponding to the two agents, following the Neuro-SERKET framework (Taniguchi et al., 2020). For clarity, consider the following variables:

• *: denotes a generic agent in the model, where * ∈ {*A*, *B*}.
• *w*_*d*_: represents a sign, such as a name, associated with the *d*th object.
• $z_{d}^{*}$ : refers to the perceptual state corresponding or the internal representation of the *d*th object associated with agent *.
• $o_{d}^{*}$ : signifies the observation of the *d*th object from the agent *.
• ϕ*: contains parameters governing the relationship between signs and their perceptual states.
• θ*: holds parameters describing the connections between perceptual states and observations.
• *α*: acts as a hyperparameter for ϕ*.
• β*: serves as a hyperparameter for θ*.

The inference via MH naming game involves two agents A and B. They take on roles as either a speaker (*Sp*) or a listener (*Li*). The combinations of roles are (*Sp*, *Li*) ∈ {(*A*, *B*), (*B*, *A*)}. Below, we report a breakdown of the process:

1. **Perception:** Initially, the speaker (*Sp*) and listener (*Li*) get the observations $o_{d}^{Sp}$ and $o_{d}^{Li}$ by both observing the *d*th object, then infer the perceptual states $z_{d}^{Sp}$ and $z_{d}^{Li}$ , respectively.
2. **MH communication:** the speaker (*Sp*) selects the sign $w_{d}^{Sp}$ by sampling from the posterior distribution $P\left(w_{d}|z_{d}^{Sp},\phi^{Sp}\right)$ and sends this sign to listener. The listener (*Li*) evaluates the received sign by applying the probability $r=\min\left(1,\frac{P\left(z_{d}^{Li}|\phi^{Li},w_{d}^{*}\right)}{P\left(z_{d}^{Li}|\phi^{Li},w_{d}^{Li}\right)}\right)$ , which serves as the acceptance criterion.
3. **Learning:** After the MH communication is completed for each object, the listener updates its parameters *ϕ* ^*Li*^, *θ* ^*Li*^ by sampling.
4. **Turn-taking:** The roles of speaker and listener are swapped, and the process returns to step 1.

The study by Taniguchi et al. (2023) provided a comprehensive explanation and validation of the approach, demonstrating its guaranteed convergence as an approximate decentralized Bayesian inference of shared representations $P\left(w_{d}|o_{d}^{A},o_{d}^{B}\right)$ .

However, the scenarios where the vocabulary size–number of words or signs–exceeds the actual number of data categories were not mentioned in the original work (Taniguchi et al., 2023). To address this gap, we have conducted an additional experiment, detailed in Supplementary Appendix S1 of our paper. Our findings underscore the versatility of the inter-GMM + VAE model, even in contexts where the vocabulary size surpasses the count of actual categories. In such settings, a single category might be represented by multiple signs or words, hinting at the presence of synonyms. This overparametrization allows agents a larger vocabulary than the number of input object categories. This communication mirrors human language in its capacity to categorize input data (Chaabouni et al., 2020; Dessì et al., 2021).

### 3.2 Variational autoencoder (VAE) and multimodal VAE (MVAE)

**Variational Autoencoder (VAE)** is a probabilistic generative model designed to learn a latent space representation of objects (Kingma and Welling, 2013). For a given dataset *x*, the VAE models the joint distribution *p*_*θ*_(*x*, *z*) with:

$$p_{\theta }\left(x,z\right)=p\left(z\right)p_{\theta }\left(x|z\right)$$ (1)

where *p*(*z*) is typically a standard Gaussian distribution N(0,I) . The model *p*_*θ*_(*x*|*z*) captures the probability of observing *x* given *z*, and it is implemented using a neural network with parameters *θ*. Due to the intractability of this distribution, VAE uses an approximate posterior *q*_*ϕ*_(*z*|*x*). The training process involves optimizing the Evidence Lower BOund (ELBO):

$$\text{ELBO}\left(x\right)=E_{q_{\phi }\left(z|x\right)}\left[\log p_{\theta }\left(x|z\right)\right]-\beta \text{KL}\left[q_{\phi }\left(z|x\right)\|p\left(z\right)\right]$$ (2)

With KL(*p*‖*q*) denotes the Kullback-Leibler divergence between *p* and *q*. The hyperparameter *β* is used for controlling the balance between the reconstruction and regularization terms in the ELBO. When *β* is appropriately adjusted, VAEs can achieve disentangled representations (Higgins et al., 2017).

**FIGURE 3:**
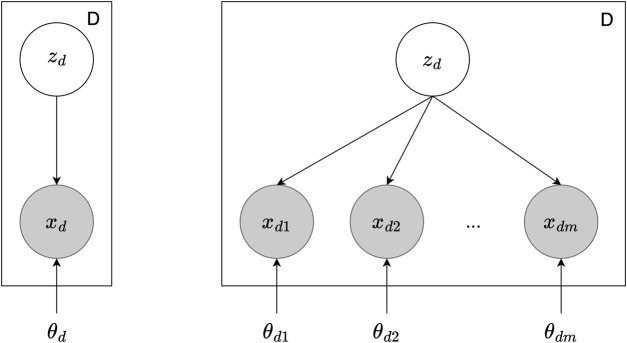
The graphical model of VAE and MVAE. On the left is the VAE with dataset *D*, latent variable *z*_*d*_ for each data point, observed data point *x*_*d*_, and the model parameters *θ*_*d*_. On the right is the MVAE with multiple data modalities. Here, *x*_*dm*_ indicates the observed data points across different modalities *m*, and *θ*_*dm*_ represent the parameters for each modality.

**Multimodal VAE:** Multimodal learning is the process of combining information from different sensory inputs to understand our surroundings. This approach is useful for artificial intelligence and robotics, as it equips models and robots with the capability to interpret their surroundings using a variety of data types (Stein and Alex Meredith, 1993; Noda et al., 2014). One of the main challenges in multimodal learning is finding a way to create a shared representation of different data types without needing explicit labels (Ngiam et al., 2011). To address this, researchers have looked into deep generative models, such as VAE Figure 3 shows the graphical model of VAE and the expansion to MVAE. These models utilize neural networks to find hidden data representations and combine these representations from multimodal data into a cohesive latent space (Suzuki and Matsuo, 2022). This area of research, focusing on deep generative models that can handle multiple types of data, has become increasingly popular in recent years (Baltrušaitis et al., 2019).

This study focuses on three common approaches for merging latent spaces: PoE-MVAE (Wu and Goodman, 2018), MoE-MVAE (Shi et al., 2019), and MoPoE-MVAE (Sutter et al., 2021). In this section, we aim to enhance the accessibility of our discussion by providing the formulations of the MVAE equations.

Consider a dataset ***X*** comprising *D* independent and identically distributed data points, denoted by $X={\left\{X_{d}\right\}}_{d=1}^{D}$ . Each data point *X*_*d*_ is characterized by a set of *M* modalities, represented by $X_{d}={\left\{x_{dm}\right\}}_{m=1}^{M}$ . Each of modality is processed by a distinct VAE to generate the corresponding latent space. To form a unified latent representation *q*(***z*** ∣*X*_*d*_) for each data point *X*_*d*_ encompassing all *M* modalities, we apply a function *f* to merge these separate latent spaces. By performing different operations on function *f*, various versions of the MVAE can be formulated (Suzuki and Matsuo, 2022):

$$q\left(z|X_{d}\right)=f\left({\left\{q_{\phi_{m}}\left(z|x_{dm}\right)\right\}}_{m=1}^{M}\right)$$ (3)

In PoE-MVAE (Wu and Goodman, 2018), each expert is trained to handle a specific aspect of the data. The final representation is obtained by multiplying the outputs from the different experts. While this focused expertise and robust integration are advantageous, it also means that the model can be disproportionately affected by poor information from one modality. The unified latent spaces is determined as follows:

$$q_{\text{PoE}}\left(z|X_{d}\right)\propto \underset{x_{dm}\in X_{d}}{\prod }q_{\phi_{m}}\left(z|x_{dm}\right)$$ (4)

In contrast, MoE-MVAE Shi et al. (2019) applies the mixture operation to learn the relationships between different data modalities. This allows for a more flexible integration of modalities and better data generation. However, this can lead to less precise representations for each modality due to diluted expert contributions. The unified latent spaces of data points are calculated as follows:

MoE-MVAE, on the other hand, applies a mixture operation,

$$q_{\text{MoE}}\left(z|X_{d}\right)\propto \underset{x_{dm}\in X_{d}}{\sum }q_{\phi_{m}}\left(z|x_{dm}\right)$$ (5)

MoPoE-MVAE (Sutter et al., 2021) combines the strengths of both approaches, PoE and MoE, and offers balanced representations. However, it also introduces increased complexity and potentially higher computational cost. In this approach, after training different experts, the final representation is obtained by multiplying the outputs from the different experts and then applying a mixture operation. Let $P\left(X_{d}\right)$ be the powerset of *X*_*d*_. The MoPoE-MVAE equation is expressed as:

$$q_{\text{MoPoE}}\left(z|X_{d}\right)\propto \underset{S\in P\left(X_{d}\right)}{\sum }q_{\mathrm{PoE}}\left(z|S\right)$$ (6)

These different methods, each with their own unique advantages and limitations, will be integrated into our model in this paper. We will then compare their performance under various conditions to evaluate their applicability.

## 4 Proposed models

### 4.1 Inter-GMM + MVAE

This section introduces the Inter-GMM + MVAE, a model designed to facilitate *EmCom* between agents and to handle multimodal data types. This model combines Gaussian mixture models (GMM), MVAE, and MH naming game in an integrative framework. The Inter-GMM + MVAE model characteristics are as follows:

• GMM forms the basis for each agent’s internal representation, clustering data points into categories. The GMM supports the agent in understanding the data categorization.
• MVAE functions as the probabilistic generative model allowing each agent to learn multiple modalities of data points or objects, such as images, sounds, and haptic sensations.
• The prefix “Inter,” derived from the Latin word for “between,” highlights the interactions and relationships between agents. This reflects concepts like “interpersonal” communication in the MH naming game.

#### 4.1.1 Model components

Assume that we have an observed dataset ***X*** of *D* independent and identically distributed data, represented as $X={\left\{X_{d}\right\}}_{d=1}^{D}$ . Each data point, *X*_*d*_, consists of a set of *M* modalities belonging to one of *K* categories, denoted as $X_{d}={\left\{x_{dm}\right\}}_{m=1}^{M}$ .

**FIGURE 4:**
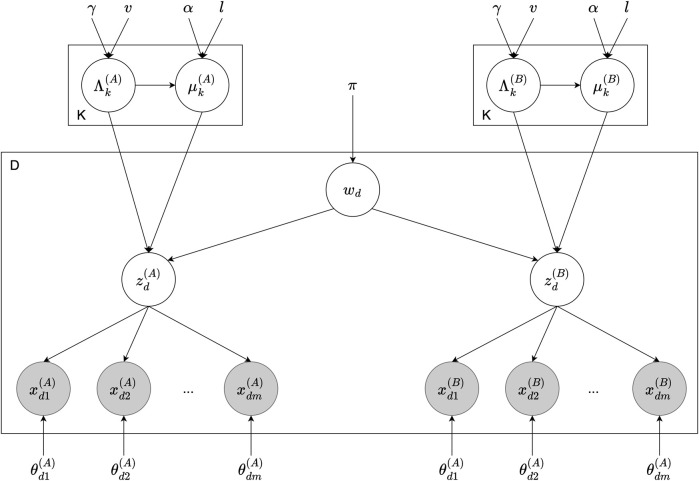
Graphical model of the Inter-GMM + MVAE model involving two agents, *A* and *B*. For each data point *d*, the sign *w*_*d*_ is drawn from the prior *π*, which influences the latent representations $z_{d}^{\left(A\right)}$ and $z_{d}^{\left(B\right)}$ corresponding to agents A and B. These latent states are organized by each agent’s GMM comprising *K* components with hyperparameters *α*, *l*, *γ*, *v*, characterized by means $\mu_{k}^{\left(A\right)}$ and $\mu_{k}^{\left(B\right)}$ , and precision matrices $\Lambda_{k}^{\left(A\right)}$ and $\Lambda_{k}^{\left(B\right)}$ , for each component *k* ∈ {1, …, *K*}. The latent representations then guide the generation of multi-modal observations $x_{dm}^{\left(A\right)}$ and $x_{dm}^{\left(B\right)}$ across *m* modalities, modeled by the parameters $\theta_{dm}^{\left(A\right)}$ and $\theta_{dm}^{\left(B\right)}$ .

Figure 4 illustrates the graphical model of the Inter-GMM + MVAE model, consisting of two agents, A and B, with shared vocabulary *w*_*n*_. The components of this model are as follows:

• *: denotes a generic agent in the model, where * ∈ {*A*, *B*}.
• *D*, *K*: represent the number of data points *D* and categories *K* of the dataset.
• $\mu_{k}^{\left(*\right)}$ , $\Lambda_{k}^{\left(*\right)}$ : refer to the mean and precision matrix of the *k*th multivariate normal distribution within the GMM component for agent*.
• *w*_*d*_: indicates the category or sign associated with the data point *X*_*d*_.
• (*α*, *l*), (*γ*, *v*), *π*: are hyper-parameters for $\mu_{k}^{\left(*\right)}$ , $\Lambda_{k}^{\left(*\right)}$ and *w*_*d*_, respectively.
• $z_{d}^{\left(*\right)}$ : signifies latent variable of MVAE for the agent (*) corresponding to data point *X*_*d*_.
• $x_{dm}^{\left(*\right)}$ , $\theta_{dm}^{\left(*\right)}$ : convey the observed information and its parameters with modality *m* of the MVAE for the agent (*) related to data point *X*_*d*_.

#### 4.1.2 Connections among modules

**FIGURE 5:**
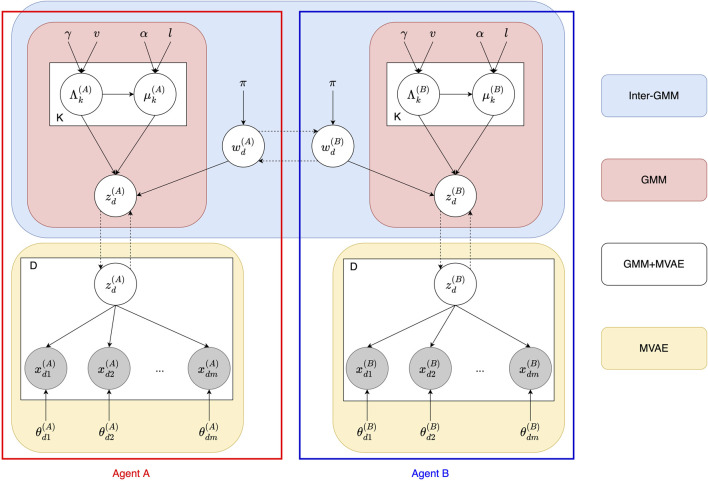
The decomposition of the Inter-GMM + MVAE model between Agents A and B. The reds represent the GMM components. The blue is the Inter-GMM that facilitates interaction between the agents via the MH naming game. The yellow are the MVAE of each agent.

To facilitate understanding of the model, Figure 5 demonstrates the decomposition of the graphical model into separate modules. The implementation combines several modules that work collaboratively to create a cohesive system capable of managing multimodal data and communication through the MH naming game. The relationships between these modules are based on the SERKET (Nakamura et al., 2018) and Neuro-SERKET (Taniguchi et al., 2020) frameworks, which aims to integrate multiple stochastic models or modules into a unified cognitive model. These frameworks are based on the concept that the brain processes information by combining of bottom-up and top-down approaches, which can be represented using probabilistic generative models. The Inter-GMM + MVAE model consists of two primary connections, as described below:

• GMM + MVAE: Connecting and combining the GMM of internal representations with the MVAE of the deep learning process for objects ensures that the GMM will structure the latent spaces representing objects received from MVAE. The notation “+” signifies the composition of two graphical models and their mutual (or simultaneous) inference, following the convention of the Neuro-SERKET framework.
• Inter-GMM: This connection represents a tail-to-tail composition of two GMMs. It is created through inference via the MH naming game between two agents, allowing them to share and update their internal representations, fostering a shared understanding of the objects.

By integrating these modules and connections, the Inter-GMM + MVAE model provides a suitable approach for handling multimodal data and promoting communication between agents.

#### 4.1.3 Inference via Metropolis-Hastings naming game

*EmCom* in the Inter-GMM + MVAE model, based on the MH naming game, involves two agents A and B. These agents alternate between the roles of speaker (Sp) and listener (Li) during their interactions, pairings of (*Sp*, *Li*) ∈ {(*A*, *B*), (*B*, *A*)}. The communication between the agents and the observed object is probabilistic. The speaker (*Sp*) perceives the object and assigns a name to it, which is selected probabilistically based on its internal state inferred from the observation. This name, represented by the word *w*, is determined by sampling from the posterior distribution of words and is then communicated to the listener (*Li*). The listener (*Li*) then decides whether to accept the word according to its belief state and calculates the judgment ratio using the MH algorithm. Subsequently, the agents switch roles or alternate turns. This process comprises the following steps:

1. **Perception:** The speaker (*Sp*) perceives the multimodal data $X_{d}={\left\{x_{dm}\right\}}_{m=1}^{M}$ . This perception is represented as *z*_*d*_ and is organized within a GMM with *μ* ^*Sp*^ and Λ^*Sp*^.
2. **Naming:** The speaker (*Sp*) samples its word *w*_*d*_ from $P\left(w_{d}|z_{d}^{Sp},\mu^{Sp},\Lambda^{Sp}\right)$ and sends the word *w*_*d*_ of that object to the listener (*Li*).
3. **MH communication:** The listener (*Li*) receives the proposed word *w*_*d*_ and decides whether to accept it using the MH algorithm. The acceptance probability is computed as $r=\min\left(1,\frac{P\left(z_{d}^{Li}|\mu^{Li},\Lambda^{Li},w_{d}^{Sp}\right)}{P\left(z_{d}^{Li}|\mu^{Li},\Lambda^{Li},w_{d}^{Li}\right)}\right)$

4. **Learning:** Based on the decision, the listener (*Li*) updates its parameters *μ* ^*Li*^ and Λ^*Li*^.
5. **Turn taking:** The roles of the speaker (*Sp*) and listener (*Li*) are swapped, and the process iterates from steps (1) through (4).

#### 4.1.4 Probabilistic generative process

We provide a clear understanding of the underlying mechanisms driving the model’s behavior and gain insights into how the model integrates information and learns to generate communication strategies. We denote * ∈ {*A*, *B*} as the agent. The probabilistic generative process of this model is described as follows:

$$w_{d}∼\text{Cat}\left(\pi \right)d=1,\ldots ,D$$ (7)

$$\mu_{k}^{\left(*\right)},\Lambda_{k}^{\left(*\right)}∼N\left(\mu_{k}^{\left(*\right)}|l,{\left(\alpha \Lambda_{k}^{\left(*\right)}\right)}^{-1}\right)W\left(\Lambda_{k}^{\left(*\right)}|v,\gamma \right)k=1,\ldots ,K$$ (8)

$$z_{d}^{\left(*\right)}∼N\left.\left(z_{d}^{\left(*\right)}|\mu_{w_{d}}^{\left(*\right)},{\left(\Lambda_{w_{d}}^{\left(*\right)}\right)}^{-1}\right)\right)d=1,\ldots ,D$$ (9)

$$x_{dm}^{\left(*\right)}∼p_{\theta_{dm}^{\left(*\right)}}\left(x_{dm}^{\left(*\right)}|z_{d}^{\left(*\right)}\right)d=1,\ldots ,D\text{ and }m=1,\ldots ,M$$ (10)

First, for *d* = 1, …, *D*, the word *w*_*d*_, which represents the shared vocabulary of agents A and B, is drawn from a categorical distribution with parameter *π*. Then, for *k* = 1, …, *K*, the mean vector $\mu_{k}^{\left(*\right)}$ and precision matrix $\Lambda_{k}^{\left(*\right)}$ , which correspond to the parameters of the *k*th multivariate normal distribution for a given agent (*), are drawn from a joint Gaussian-Wishart distribution with hyperparameters *α*, *l*, *γ* and *v*. Next, the latent variable $z_{d}^{\left(*\right)}$ representing the latent space of the MVAE for agent (*), is drawn from a multivariate normal distribution with mean vector $\mu_{w_{d}}^{\left(*\right)}$ and covariance matrix ${\left(\Lambda_{w_{d}}^{\left(*\right)}\right)}^{-1}$ . This latent variable captures the underlying structure of the data in a lower-dimensional space, with categorical assignments provided by GMM. Finally, for *m* = 1, …, *M*, the observed information $x_{dm}^{\left(*\right)}$ is generated using the corresponding parameters $\theta_{dm}^{\left(*\right)}$ .

Following this generative process, the Inter-GMM + MVAE model can capture the underlying structure of the multimodal data. The model considers the categorical assignments using GMM and comprehends the relationships between different modalities.

Algorithm 1 Inference via MH naming game

**   **Initialize Agent A and Agent B
**   while **(mutual iteration) **do**
**   ** Train MVAE of Agent A and Agent B
**   ** while** **(MH-learning iteration) **do**
**   ** MH algorithm(from A to B)
**   ** Update parameters of Agent B
**   ** MH algorithm(from B to A)
**   ** Update parameters of Agent A
**   ** end **while**
** end while**

#### 4.1.5 Running procedure

This iterative process facilitates communication and understanding between the two agents, allowing them to develop a shared understanding of the environment and categories of multimodal objects. The pseudo-code for this system is given in Algorithm 1 and can be described as follows:

• Initially, agents A and B are set up with their respective data loaders and model parameters.
• The mutual iteration loop encompasses the entire communication process between the agents, including training the multimodal VAE of both agents.
• Inside the mutual iteration loop, a nested loop (MH-learning iteration) focuses on the communication process of the two agents via the MH algorithm.
• At first, agent A acts as the speaker, sharing its understanding of the data points using the MH algorithm. Agent B, acting as the listener, then receives this information and updates its parameters accordingly, refining its internal model of the data.
• Subsequently, the roles are reversed: Agent B becomes the speaker and agent A the listener. Using the MH algorithm, agent B communicates its understanding to agent A, which then updates its parameters.
• This MH-learning iteration loop continues until a certain condition, such as convergence or the maximum number of iterations, is satisfied.
• The entire mutual iteration loop also proceeds until a stopping criterion is reached. Throughout this process, the agents learn from each other using the MH naming game, update their parameters, and refine their internal models of the shared data points.

### 4.2 Inter-GMM + Weighted-*β*-MVAE

The Inter-GMM + Weighted-*β*-MVAE model is an extension of the Inter-GMM + MVAE model. It is designed to balance two key elements: the contributions of different modalities in multimodal data and the balance between data reconstruction and latent space regularization (controlled by parameter *β*). Certain modalities may be more structured or simpler to interpret in any dataset. Therefore, focusing on these modalities can improve the communication process between agents in *EmCom*. This approach mirrors human interactions and conversations, where explanations focus on more readily understood concepts, facilitating “easier” comprehension by listeners.

In addition, the *β* parameter influences the degree of disentanglement in the latent space. This parameter mediates between two competing objectives: minimizing the reconstruction loss and encouraging the disentanglement of latent spaces by scaling the Kullback-Leibler (KL) divergence term. Disentangled representations enable each dimension in the latent space to correspond to distinct interpretable variation factors in a dataset (Higgins et al., 2017). In *EmCom*, such clear internal representations assist agents in aligning their vocabularies and promoting a shared understanding for successful communication.

Let ***X*** denote the observed dataset with *D* independent and identically distributed data points, represented as $X={\left\{X_{d}\right\}}_{d=1}^{D}$ . Each data *X*_*d*_ comprises of a set of *M* modalities, denoted as $X_{d}={\left\{x_{dm}\right\}}_{m=1}^{M}$ .

The loss function for each data point in the Inter-GMM + Weighted-*β*-MVAE model is as follows:

$$\begin{array}{ll} L_{E}\left(X_{d}\right) & =-\beta \text{KL}\left(q_{E}\left(z|X_{d}\right)\|p\left(z\right)\right)\\ & \;+\underset{x_{dm}\in X_{d}}{\sum }\lambda_{m}E_{q_{E}\left(z|X_{d}\right)}\left[\log p_{\theta_{dm}}\left(x_{dm}|z\right)\right] \end{array}$$ (11)

where

• *E*: denotes the expert operations (MoE, PoE, MoPoE) (described in Section 3.2) used to aggregate the individual latent spaces derived from each modality into a joint latent space.
• *z*: represents latent space of the data from all modalities.
• *p*(*z*): signifies prior distribution of the latent variable *z*, assumed to be a standard multivariate Gaussian distribution.
• *q*_*E*_(*z*∣*X*_*d*_): indicates the approximated posterior distribution of *z* given the observed data *X*_*d*_.
• *θ*_*dm*_: embodies model parameters associated with the *m*th modality of data *X*_*d*_.
• $p_{\theta_{dm}}\left(x_{dm}|z\right)$ : is the likelihood of the observed data *x*_*dm*_ (the *m*th modality of data *X*_*d*_), given the latent variable *z* and parameterized by *θ*_*dm*_.
• *β*: corresponds to the parameter controlling the weight of the KL divergence term, thus determining the degree of disentanglement in the latent space.
• *λ*_*m*_: conveys the weight assigned to each modality *m* in the data *X*_*d*_, indicating its importance in the overall data reconstruction.

The loss function in Eq. 11 comprises two main terms:

• The first is the Kullback-Leibler (KL) divergence between the approximated posterior distribution *q*_*E*_(*z*∣*X*_*d*_) and prior distribution *p*(*z*), which is typically assumed to be a standard multivariate Gaussian distribution. This term, scaled by a factor *β*, encourages the model to learn a disentangled latent space by minimizing the difference between the approximated posterior and the prior. The *β* parameter controls the trade-off between disentangling and reconstruction, with higher values emphasizing a more disentangled latent space.
• The second corresponds to the reconstruction loss. It is the sum of the expected log-likelihood of each observed modality *x*_*dm*_ in data *X*_*d*_ given the latent variable *z*. This expectation concerns the approximated posterior distribution *q*_*E*_(*z*∣*X*_*d*_). Specifically, the computation of *z* depends on the expert operation *E* (MoE, PoE, or MoPoE), aggregating the individual latent representations derived from each modality into a joint latent space. This term measures how well the model reconstructs the observed data from the latent variable *z*. Furthermore, each modality is weighted by a factor *λ*_*m*_, representing the importance of that specific modality in the overall reconstruction. The model can prioritize reconstructing specific modalities over others by multiplying the expected log-likelihood for each modality with this weight.

Thus, the first term encourages the model to learn a disentangled latent space close to the prior, and the second term encourages the model to learn a latent space with the likely observed data given the latent variables. The balance between these two competing objectives is controlled by *β* and the modality weights *λ*_*m*_.

### 4.3 Hyper-parameter tuning

During the experimental process, we observed that certain datasets presented unique challenges during the model training. For instance, expert operations can cause the latent space to become overly concentrated or flattened at unexpected locations. This irregular distribution of the latent space could prevent GMM from functioning optimally, primarily because of a mismatch between the predefined hyperparameters and the actual latent space configurations, leading to inaccurate results.

To address these challenges, we propose dynamic adjustment of the hyperparameters during training. This strategy aims to shift the coordinates of the latent space to optimal positions. Specifically, we calculate the means of all the data points in the latent space and determine the new coordinates for the updated latent space based on these means. This adjustment allows the stochastic process of GMM to adapt to new hyperparameters, facilitating a more balanced distribution in the latent space. The primary objective of this strategy is to improve the accuracy of the stochastic points generated during training, thereby enhancing the overall model performance.

The pseudo-code for the Inter-GMM + Weighted-*β*-MVAE models, including the hyperparameter tuning strategy, is detailed in Algorithm 2. This procedure is similar to that outlined in Algorithm 1 and described in Section 4.1.5. The key distinction is that the hyperparameters *l* and *v*, which are part of the set of hyperparameters (*α*, *l*), (*γ*, *v*), respectively, associated with each agent (described in Section 4.1.1 and illustrated in Figure 4), are dynamically adjusted following the MVAE training during each round of mutual iteration. This process ensures continuous alignment of the hyperparameters with evolving configurations of the latent space throughout training.

Algorithm 2 Inter-GMM + MVAE with hyperparameter tuning

**   **Initialize Agent A and Agent B
**   while **(mutual iteration)** do**
**    for **(each agent) **do**
**   ** Train MVAE
**   ** Compute the mean and variance of all data points in latent space
**   ** Update the agent’s hyperparameters *l* and *v*
**    end for**
**    while **(MH-learning iteration) **do**
**   ** MH algorithm(from A to B)
**   ** Update parameters of Agent B
**   ** MH algorithm(from B to A)
**   ** Update parameters of Agent A
**    end while**
** end while**

## 5 Experiment 1: MNIST + SVHN

This experiment aims to evaluate the proposed model under the conditions posited by our experimental hypotheses (see Section 5.1). We used the benchmark MNIST (LeCun et al., 2010) and SVHN (Netzer et al., 2011) datasets because they provide a controlled environment that simulates multimodal sensory data, making them ideal for this evaluation.

### 5.1 Hypotheses

Addressing the three questions outlined in the Introduction 1, this section puts forth three hypotheses concerning the proposed Inter-GMM-MVAE model, as follows:

(1) By integrating the MH naming game with MVAE, agents can establish perceptual categories and communicate signs using multimodal sensory data.
(2) Even when some modalities are missing, semiotic communication between agents allows for continued categorization accuracy within the agent.
(3) Increasing the weight ratio to emphasize a more readily modality improves agent communication.

### 5.2 Datasets

We constructed a multimodal dataset by pairing the MNIST (LeCun et al., 2010) and SVHN (Netzer et al., 2011) datasets, comprising images of handwritten digits and street-view house numbers, respectively. Each data point in our combined dataset consists of an MNIST image and a corresponding SVHN image representing a single digit. Though both datasets represent numerical digits, they are inherently different in style and visual context. We term this combined dataset “multimodal”, following the convention in multimodal VAE research where different representations of similar data are integrated, not in the sense of different data types (e.g., audio and visual), but rather in the sense of integrating different visual representations.

**FIGURE 6:**
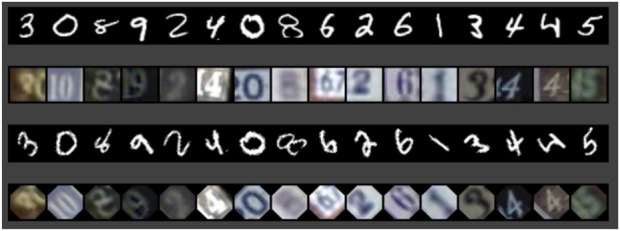
One object consists of two images (MNIST + SVHN) of one digit. Agent A with original images (rows 1 and 2). Agent B with images rotated 45° (rows 3 and 4)

We assigned the dataset to two agents, A and B. Agent A received the original MNIST and SVHN images, whereas agent B received versions of the same images rotated by 45° (Figure 6). This design choice allowed us to evaluate the capability of the model to vary the input data and assess the agents’ ability to establish a shared understanding despite these differences. Such an arrangement simulates the inherent differences in perspective that characterize human communication, where no two individuals experience the same scene in an identical manner. Our approach is in line with data augmentation previously investigated in (Dessì et al., 2021; Kharitonov et al., 2021).

### 5.3 Conditions

To ensure a fair comparison, the following evaluation metrics were consistently applied across all experimental scenarios: Cohen’s Kappa coefficient (Kappa) (Cohen, 1960), Adjusted Rand Index (ARI) (Hubert and Arabie, 1985), Davies-Bouldin Score (DBS) (Davies and Bouldin, 1979) and Fréchet Inception Distance (FID) (Heusel et al., 2017). More details about these metrics can be found in Supplementary Appendix S2. Furthermore, we use t-distributed Stochastic Neighbor Embedding (t-SNE) (van der Maaten and Hinton, 2008), a dimensionality reduction technique, to visualize each agent’s internal representations (latent spaces). These visualizations assist in understanding the structure and distribution of the latent spaces and interpreting the results from our evaluation metrics.

A direct comparison between our models and other methods is impractical because of the unique integration of the MH naming game and MVAE in our approach, which fundamentally differs from existing models. Instead, we evaluate our models under various conditions designed to test our experimental hypotheses:

#### 5.3.1 Condition 1

To validate hypothesis (1), we assess the performance of Inter-GMM + MVAE under two contrasting baseline conditions: “All accepted” and “No communication.”

• All accepted: Agents approve all messages without evaluation. This allows us to measure the impact of unrestricted information acceptance on multimodal learning.
• No communication: Agents do not communicate. This scenario allows us to understand the role of communication in multimodal learning within our MVAE models.

#### 5.3.2 Condition 2

To evaluate hypothesis (2), we investigate the performance of Inter-GMM + MVAE when certain modalities are missing. This evaluates how communication between agents can compensate for missing sensory data. The specific scenarios considered are as follows:

• Full Modality Scenario: Both agents have full access to the MNIST and SVHN datasets.
• Scenario 2: Agent A has full access to both modalities, while Agent B has access only to MNIST.
• Scenario 3: Agent A has full access to both modalities, but Agent B has access only to SVHN.

#### 5.3.3 Condition 3

To evaluate hypothesis (3), we adjust the weight ratio between the MNIST and SVHN modalities and vary *β* parameter that governs disentangling in the latent space. This will help us understand how prioritizing a more distinct modality affects communication between agents. The specific models compared are as follows:

• MNIST:SVHN = 1:1, *β* = 1. This is our baseline model, with equal dataset weights and *β* = 1.
• MNIST:SVHN = 1:1, *β* = 100. Model with increased *β* to assess its effect on model accuracy.
• MNIST:SVHN = 4:1, *β* = 1. Model with the dataset weight skewed towards MNIST.
• MNIST:SVHN = 4:1, *β* = 100. Model with the dataset weight skewed towards MNIST and increased *β*.

#### 5.3.4 Experimental setup

This experiment applied a latent dimension of 20 and consisted of three iterative stages: mutual iteration (5 times), MVAE training iteration (10 times), and the MH naming game (10 times). Three MVAE models: MoE (Inter-GMM + MoE-MVAE), PoE (Inter-GMM + PoE-MVAE), and MoPoE (Inter-GMM + MoPoE-MVAE), were tested. Variational parameter *β* values of 1 and 100, and weighted ratios between the MNIST and SVHN datasets of 1:1 and 4:1, were also examined. The model architecture is shown in Supplementary Appendix Figure S9 of Supplementary Appendix S3.

### 5.4 Experimental results

#### 5.4.1 Results in condition 1

The results are listed in Tables 1. The key observations are as follows:

• Kappa, ARI, and DBS values confirm hypothesis (1): agents can establish perceptual categories and communicate signs based on multimodal sensory data. The MH naming game improves information sharing and learning between agents of multimodal DGM.
• Furthermore, despite the impact of various configurations, the FID values are consistently high, indicating high-quality generated images, suggesting that the MH naming game primarily influences knowledge sharing and does not impact the training model.
• Moreover, multimodal VAE methods, such as MoE, PoE, and MoPoE, significantly affect the creation of latent spaces. The PoE generates the highest overall ARI values, creating meaningful latent spaces compared to MoE and MoPoE.

**TABLE 1:** The results of condition 1 with *All Accepted* and *No Communication* scenarios. In each column, the best results are denoted by underlined and bold numbers, while the second-best results are indicated by bold numbers only.

Scenarios	Kappa	ARI		DBS		FID-MNIST		FID-SVHN	
		**A**	**B**	**A**	**B**	**A**	**B**	**A**	**B**
**Inter-GMM + MoE-MVAE**									
MH naming game	**0.903**	0.051	0.052	13.10	13.82	**24.6**	**15.4**	124.1	79.9
All accepted	0.576	0.034	0.031	20.64	21.58	26.6	17.4	76.5	60.3
No communication	0.010	0.003	0.003	33.48	32.32	33.7	19.4	90.2	67.1
**Inter-GMM + PoE-MVAE**									
MH naming game	**0.953**	**0.315**	**0.314**	**4.41**	**3.62**	48.8	37.0	**86.7**	**53.6**
All accepted	0.833	0.024	0.025	3.00	2.44	41.1	39.7	85.7	55.7
No communication	0.018	0.003	0.003	29.30	29.53	51.4	40.5	115.1	106.1
**Inter-GMM + MoPoE-MVAE**									
MH naming game	0.901	**0.179**	**0.180**	**12.46**	**11.43**	**23.7**	**16.2**	**114.1**	**30.3**
All accepted	0.700	0.121	0.108	14.65	13.30	26.2	18.8	87.9	56.1
No communication	0.011	0.003	0.003	31.97	33.50	24.4	17.0	85.3	48.9

#### 5.4.2 Results in condition 2

The experimental results when investigating the influence of the missing modalities on the MH naming game are shown in Table 2. The key observations are as follows:

• The good Kappa results confirm hypothesis (2), asserting that semiotic communication between agents can sustain categorization accuracy, even in the absence of specific modalities.
• In addition, Kappa values are highest when both agents have full modalities, signifying optimal agreement when both have complete information. This highlights the significance of communication in fully-multimodal learning settings.
• ARI and DBS are highest when agent B has only MNIST, while the lowest scores occur when B has only SVHN. This indicates the more orderly structure of MNIST compared to SVHN, emphasizing that communication tends to be better with more systematically organized data.

**TABLE 2:** The results of condition 2 in *modality missing* scenarios. In each column, the best results are denoted by underlined and bold numbers, while the second-best results are indicated by bold numbers only.

Scenarios	Kappa	ARI		DBS	
		**A**	**B**	**A**	**B**
**Inter-GMM + MoE-MVAE**					
No missing modality	0.903	0.051	0.052	13.10	13.82
Agent B with only MNIST	0.825	0.494	**0.657**	20.73	2.91
Agent B with only SVHN	0.487	0.002	0.001	16.41	6.81
**Inter-GMM + PoE-MVAE**					
No missing modality	**0.954**	0.315	0.314	**4.40**	3.62
Agent B with only MNIST	**0.928**	**0.564**	0.575	5.16	**2.75**
Agent B with only SVHN	0.606	0.137	0.127	**3.18**	9.83
**Inter-GMM + MoPoE-MVAE**					
No missing modality	0.901	0.179	0.180	12.46	11.43
Agent B with only MNIST	0.883	**0.541**	**0.618**	17.30	**2.76**
Agent B with only SVHN	0.389	0.023	0.002	10.67	7.39

#### 5.4.3 Results in condition 3

The experimental results are presented in Table 3. Key insights include:

• Kappa, ARI, and FID results show that emphasized weight ratio on a distinct modality can improve agent communication and validate hypothesis (3).
• Across all models, FID values are consistently good, indicating a high-quality image generation. Notably, higher beta values negatively affect FID scores, while adjustments in dataset weight ratios have a positive impact, leading to better image generation. The generated images are shown in Supplementary Appendix Figure S11, S12, S13.
• DBS values are good, keeping stability and consistency across various configurations with no noticeable changes when adjusting weights and *β*.
• ARI values vary significantly among the models. PoE models exhibit the best ARI values overall, especially when the weight ratio is adjusted to focus on the MNIST modality.
• The t-SNE visualizations (Supplementary Appendix Figure S14) reveal that PoE models generate the most well-separated clusters, notably when the weight is skewed towards MNIST. Meanwhile, MoPoE presents moderately distinct clusters, whereas the clusters in MoE models’ visualizations are less well-defined and harder to interpret.

**TABLE 3:** Evaluation results under condition 3: Impact of varying ratios of MNIST to SVHN (denoted as r = MNIST:SVHN) and different *β* values on the model’s performance. In each column, the best results are denoted by underlined and bold numbers, while the second-best results are indicated by bold numbers only.

Scenarios		Kappa	ARI		DBS		FID-MNIST		FID-SVHN	
r	***β***		**A**	**B**	**A**	**B**	**A**	**B**	**A**	**B**
**Inter-GMM + weighted-** ***β*** **-MoE-MVAE**										
r = 1:1	*β* = 1	0.903	0.051	0.052	13.10	13.82	24.6	15.4	124.1	79.9
r = 1:1	*β* = 100	0.641	0.003	0.003	10.38	11.96	97.8	83.6	293.3	137.9
r = 4:1	*β* = 1	0.753	0.010	0.015	3.29	3.45	27.4	**14.2**	115.9	**46.6**
r = 4:1	*β* = 100	0.859	0.122	0.116	3.11	3.23	45.4	49.6	240.7	128.8
**Inter-GMM + weighted-** ***β*** **-PoE-MVAE**										
r = 1:1	*β* = 1	0.953	0.315	0.314	4.41	3.62	48.8	37.0	86.7	53.6
r = 1:1	*β* = 100	0.941	0.308	0.313	5.87	5.37	100.1	85.4	222.6	119.4
r = 4:1	*β* = 1	**0.970**	**0.794**	**0.791**	3.80	3.30	32.4	21.5	**78.2**	55.5
r = 4:1	*β* = 100	**0.972**	**0.713**	**0.710**	3.53	3.32	45.0	32.6	251.9	151.9
**Inter-GMM + weighted-** ***β*** **-MoPoE-MVAE**										
r = 1:1	*β* = 1	0.901	0.179	0.180	12.46	11.43	**23.7**	16.2	114.1	**30.3**
r = 1:1	*β* = 100	0.892	0.473	0.477	**2.60**	**2.54**	96.1	71.7	227.1	101.3
r = 4:1	*β* = 1	0.894	0.344	0.358	15.77	12.31	**23.2**	**13.6**	**85.7**	52.8
r = 4:1	*β* = 100	0.889	0.230	0.233	**2.29**	**2.18**	56.2	45.5	246.5	102.1

Our experiments confirmed three key hypotheses: First, integrating MH naming game and MVAE enables agents to establish perceptual categories and communicate signs for multimodal data. Second, even with missing modalities, semiotic communication between agents sustains categorization accuracy within an agent. Third, emphasizing a more distinct modality by increasing the weight ratio improves agent communication.

Furthermore, our experiments revealed the unique characteristics of different models. The MoE is suitable for image generation; however, its application in the MH naming game encounters difficulties. The MoPoE, with the potential for *EmCom*, could benefit from further refinement. In contrast, the PoE consistently yielded the best results. The Inter-GMM + PoE-MVAE proved to be a potent tool for symbol emergence with multimodal DGM and MH naming game, as evidenced by high Kappa scores, commendable ARI scores, and stable DBS values.

## 6 Experiment 2: Multimodal165

In experiment 2, we employed the Inter-GMM + MVAE model to examine hypotheses (1) and (3) (as described in Section 5.1) using a real-life object dataset. During implementation, we observed that the latent spaces from the real-life object datasets were less organized and more divergent than those from the benchmark datasets. This divergence caused the GMM to struggle to capture the latent space structure accurately, leading to errors in training and reduced performance. Therefore, we adjusted certain hyperparameters during training (as described in Section 4.3) to accurately identify the latent space coordinates using the newly calculated means of all data points.

### 6.1 Dataset

**FIGURE 7:**
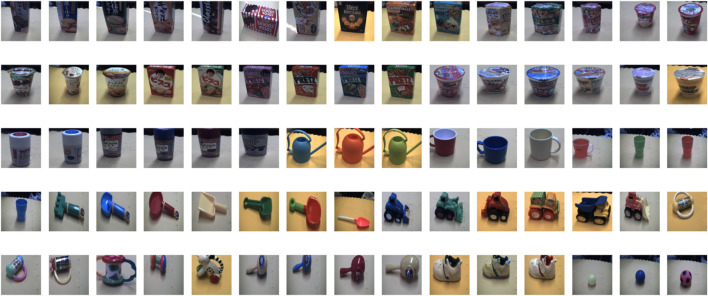
Samples from Multimodal165 - real-life object dataset.

The Multimodal165 dataset, developed by Nakamura et al. (Nakamura and Nagai, 2018), comprises 165 directories, each containing robot-generated multimodal data for a specific object. This data includes visual, auditory, haptic, and word information obtained by observing, grasping, shaking, and describing objects (Figure 7).

In this experiment, the agents were assumed to learn about real-life objects autonomously, without human input, by focusing on visual, audio, and haptic modalities. For vision, images were resized to 256 × 256 × 3, with agent A receiving a −30-degree angle image, while agent B obtaining a 0-degree angle image, providing distinct perspectives. For audio, shaking sounds were processed using MFCC = 20; agent A obtained sound lengths from 0 to 4/5 of the recording, while agent B used from 1/5 to the end, giving each agent unique audio data. For haptic data, A and B access the first and second grasping haptic information, respectively, enabling them to experience different haptic aspects of the objects.

### 6.2 Conditions

Each data instance has three modalities: visual, audio, and haptic. The latent dimension, that is, the number of dimensions in the abstract space where the data are represented, was set to 21. This selection of a 21-dimensional latent space is based on the dataset architecture. Given the three modalities of the experiment, we choose a latent space whose dimensionality is divisible by three, facilitating the integration and combination with MoE and MoPoE models, which necessitate dimensions that are multiples of three.

In this experiment, we evaluate three variations of the Inter-GMM+(MoE/PoE/MoPoE)-MVAE model. Each variant will undergo examination in three contexts: the baseline Inter-GMM + MVAE, an iteration with hyperparameter tuning, and a version with both hyperparameter tuning and a weight adjustment across the modalities. Because the haptic modality is the best-organized modality within the visual, audio, and haptic modalities in our dataset, the weighting will be biased in favor of the haptic modality at a ratio of “r = visual:audio:haptic = 1:1:3”.

The experiment consists of 10 mutual iterations (including iterations between models), 30 MVAE training iterations, and 30 MH naming game iterations. The model architecture is displayed in Supplementary Appendix Figure S10 of Supplementary Appendix S3.

### 6.3 Experimental results

Table 4 compares the results of the three models. Key findings include the following:

• Kappa values validate the first hypothesis for the Inter-GMM + MVAE with a real-world three-modality (vision, audio, and haptic) object dataset. This outcome demonstrates that the integrated MH naming game with MVAE allows agents to form perceptual categories and devise communication signs derived from multimodal data.
• The dynamic strategic adjustment of hyperparameters, based on the calculated means of all data points in the latent space, substantially enhances Kappa and DBS. This hyperparameter tuning strategy optimizes data distribution of data in the latent space, aligning it better with the Gaussian Mixture Model (GMM), and improves agreement among agents and clustering quality in the MH naming game. Despite this, consistently low ARI values suggest that these adjustments have not significantly enhanced clustering agreement with true labels.
• Concentrating on the best-organized modality led to a notable increase in ARI. Therefore, these results have validated hypothesis (3) that amplifying the weight ratio to emphasize a more differentiated modality leads to better agent communication.
• The findings further indicates that the Inter-GMM + MVAE model utilizing the PoE achieved the best results overall.

**TABLE 4:** The comparison of Inter-GMM + weighted-*β*-PoE-MVAE in Multimodal165 dataset. Here, r represents the weight adjustment and “Hyper.” indicates the hyperparameter tuning strategy. In each column, the best results are denoted by underlined and bold numbers, while the second-best results are indicated by bold numbers only.

Scenarios		*Kappa*	ARI		DBS	
r	Hyper		*A*	*B*	*A*	*B*
**Inter-GMM + weighted-** ***β*** **-MoE-MVAE**						
*No*	*No*	0.219	0.001	0.002	13.20	16.53
*No*	*✓*	0.782	0.012	0.011	2.45	3.64
*✓*	*✓*	0.869	0.414	0.402	1.58	2.89
**Inter-GMM + weighted-** ***β*** **-PoE-MVAE**						
*No*	*No*	0.215	0.010	0.002	20.49	10.58
*No*	*✓*	0.883	0.026	0.028	$\underline{0.12}$	$\underline{0.26}$
*✓*	*✓*	$\underline{0.894}$	$\underline{0.570}$	$\underline{0.570}$	0.14	0.58
**Inter-GMM + weighted-** ***β*** **-MoPoE-MVAE**						
*No*	*No*	0.211	0.001	0.002	25.31	23.50
*No*	*✓*	0.856	0.009	0.014	3.52	3.58
*✓*	*✓*	0.874	0.315	0.430	1.94	2.68

In general, this experiment demonstrated that the Inter-GMM + weighted-*β*-MVAE model is suitable for real-life object datasets, especially the model with the PoE implementation. With further improvements, these models can become even more accurate.

## 7 Conclusion

The study explored the implementation of multimodal deep generative models in *EmCom* systems within environments that focus on joint attention where both speaker and listener are aware of the same object. Our primary objective was to enable agents to process multimodal data from various sources, such as images, text, and audio, and to integrate this information into a cohesive representation. Building on the foundation of Inter-GMM + VAE (Taniguchi et al., 2023), we obtain the following results:

(1) We successfully extended the Inter-GMM + VAE model by integrating a multimodal DGM for symbol emergence based on multimodal data. Our novel model, Inter-GMM + MVAE, demonstrates that integrating the MH naming game with multimodal VAE can aid agents in constructing perceptual categories and communicating signs derived from multimodal sensory inputs.
(2) Our proposed model maintains the categorization function of each agent via semiotic communication, even when specific modalities are absent.
(3) Improving the weight ratio to highlight a modality more readily learned and categorized by the agent can improve *EmCom*. This approach mirrors human communication dynamics, where emphasis on more readily understood concepts enhances comprehension by listeners.

This study examined three multimodal techniques, MoE, PoE, and MoPoE, refining these models by factoring in the weight of each modality in the multimodal VAE and adjusting the *β* value to disentangle the latent space. Furthermore, the experiments showed that the MH naming game primarily influenced information sharing and knowledge formation without significantly affecting the training model, leading to high-quality generated images across all scenarios. However, the combination of multimodal VAE methods, such as MoE, PoE, and MoPoE, significantly affects the creation of latent spaces. Whereas MoE performed best in terms of image generation quality, PoE generated the highest overall ARI values in creating meaningful latent spaces.

In addition, our experiments with real-life datasets highlight a limitation in the current model’s ability to represent real-world objects accurately. To address this issue, we implemented a latent space coordinate refinement strategy. This approach optimizes the positioning of coordinates in the latent space by calculating the means of all data points. This hyperparameter tuning strategy considerably enhanced the sign-sharing agreement by adjusting the latent space coordinator, improving agent agreement and clustering quality within the MH naming game.

In conclusion, the integration of the MH naming game with a multimodal VAE offers considerable advancement in the field of *EmCom*. By employing PoE, MoE or MoPoE for MVAE, along with weight ratio and *β* adjustments, the experiments showed that Inter-GMM + weighted-*β*-MVAE with PoE could create better results than the model with MoE and MoPoE.

Additionally, this study includes experiments to evaluate the Inter-GMM + VAE when the vocabulary size exceeds the actual number of data categories, an aspect not explored in the original work (Taniguchi et al., 2023). The findings presented in Supplementary Appendix S1 reveal that the performance of the Inter-GMM + VAE model is sustained even with a vocabulary size greater than the number of categories in the dataset. This condition permits the assignment of several signs or words to a single category, indicative of the potential for synonyms in the communication system of the agents.

In the future, it is possible to advance the field of *EmCom* systems further. The first initiative would involve expanding the Inter-GMM + MVAE framework from a two-agent naming game to scenarios involving three or more agents by applying the method proposed in (Inukai et al., 2023). Moreover, the exploration of alternative DGM within the Inter-GMM + MVAE could present valuable options besides the currently utilized multimodal VAE. There is also consideration of transitioning from using the Gaussian Mixture Model to implementing neural networks for the learning of internal representations and word formation within the Inter-GMM + MVAE model. Such an adaptation could lead to the development of more versatile communication systems where agents are capable of conveying compositional messages, thereby enhancing the efficacy of information exchange, knowledge construction, and data reconstruction in*EmCom* contexts.

## Data Availability

For the first experiment: the MNIST database of handwritten digits is publicly available and can be accessed at LeCun et al. (2010). The Street View House Numbers (SVHN) dataset is also publicly available and can be accessed at Netzer et al. (2011). These datasets are widely used for training and testing in the field of machine learning and are freely available for educational and research purposes. For the second experiment: the Multimodal165 dataset was initially created in Nakamura and Nagai (2018). This dataset is available upon reasonable request to the original authors. For access, please refer to the contact information provided in the cited work.
