# Supplementary material for: Emergent communication of multimodal deep generative models based on Metropolis-Hastings naming game
Source: Front Robot AI. 2024 Jan 31;10:1290604. doi: 10.3389/frobt.2023.1290604 (PMC10864618; doi:10.3389/frobt.2023.1290604)
Supplement: Supplementary file 1 [file DataSheet1.pdf]

Emergent Communication of Multimodal Deep Generative Models based on Metropolis-Hastings Naming Game

Nguyen Le Hoang, Tadahiro Taniguchi, Yoshinobu Hagiwara, and Akira Taniguchi

1 Appendix 1 - Inter-GMM+VAE with larger vocabulary size

The original study [Taniguchi et al., 2023] did not address scenarios in which the vocabulary size—the number of words or signs—surpasses the actual number of data classes. In this appendix, we explore this aspect by conducting additional experiments under identical conditions to the original study. The specifics are as follows:

We utilize the Inter-GMM+VAE model. The experiments are carried out on the MNIST dataset. Agent A's observations consisted of unaltered MNIST images, while Agent B observed MNIST images rotated by 45 degrees to the left. We employ a straightforward configuration of convolutional and deconvolutional networks, each with three layers for the respective encoders and decoders within the VAE framework. We set the latent space dimension size to 12. The process includes 100 MH iterations and 100 VAE iterations. We use the baseline with the vocabulary size = 10 (matching the number of MNIST categories) and then increase this size to 20, 50, and 100 to study the effects of a vocabulary size that exceeds the number of actual categories.

The model's performance is evaluated using Kappa, DBS, and FID metrics (more details in Appendix 2), complemented by t-SNE visualizations for a graphical representation of the results. The results are displayed in Table 1, and the t-SNE visualizations are shown in Figure 1. The findings indicate that although there is a slight decline in the Kappa score with an increased vocabulary size, the score remains within the bounds of "almost perfect agreement." Furthermore, the DBS and FID values demonstrate consistency and remain comparably close to one another, regardless of the variations in vocabulary size.

In conclusion, our experiments reveal that the Inter-GMM+VAE model maintains its performance when the vocabulary size is larger than the number of categories present in the data. This scenario allows for multiple signs or words to represent a single category, suggesting the emergence of synonym-like representations in the agents' communication. This overparametrization allows agents a larger vocabulary than the number of input object categories. This communication mirrors human language in its capacity to categorize input data.

**Voca. Size**	**Kappa**	**DBS A**	**DBS B**	**FID A**	**FID B**
10	0.954	2.363	2.424	0.042	0.038
20	0.936	2.258	2.283	0.036	0.033
50	0.917	2.424	2.413	0.037	0.039
100	0.895	2.307	2.273	0.045	0.038

Table 1: Performance evaluation of Inter-GMM+VAE on the MNIST dataset across various vocabulary sizes using Kappa, DBS, and FID metrics. The results indicate the model's performance in handling an increase in vocabulary size.

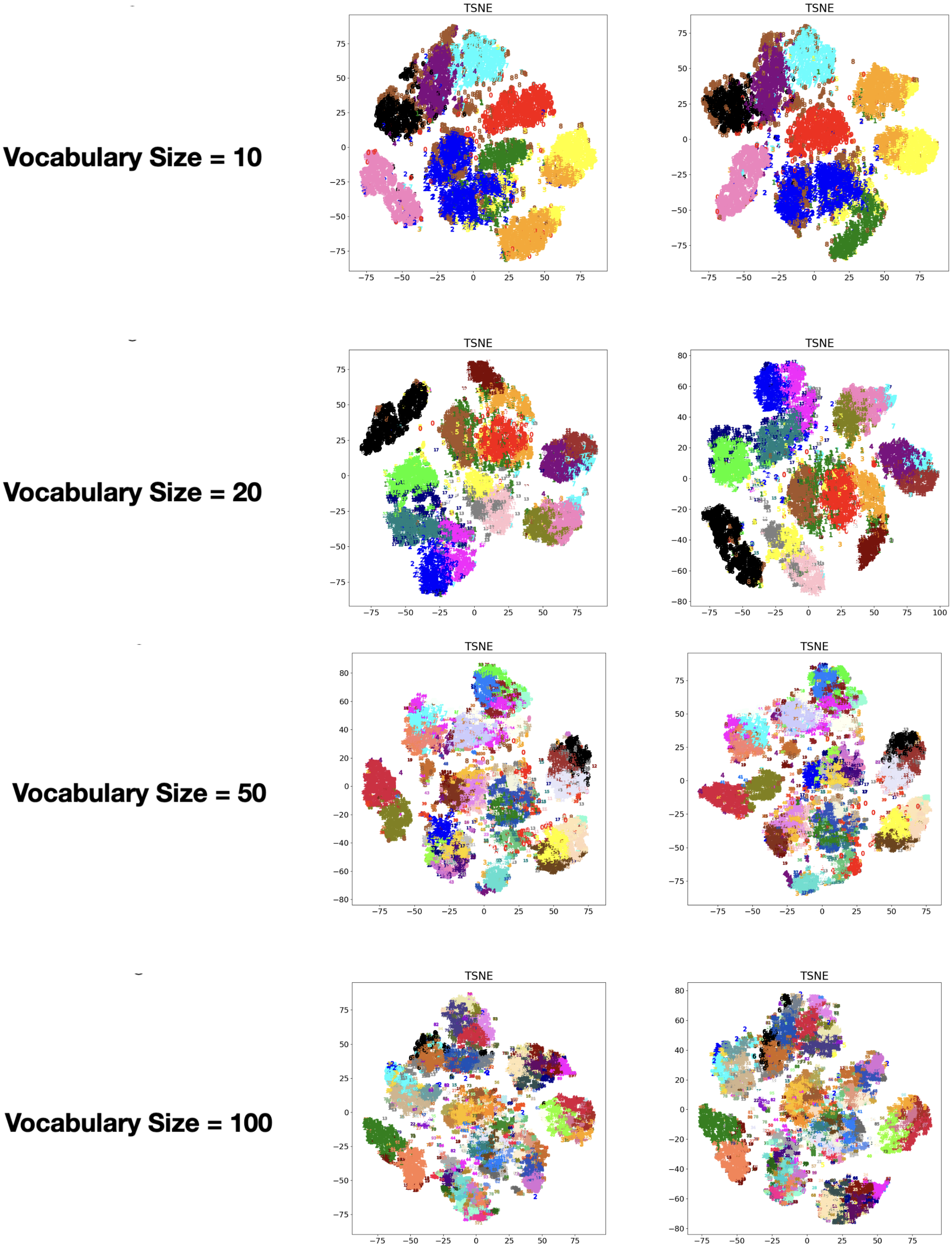

Figure 1: Visualization of t-SNE clustering for the Inter-GMM+VAE applied to the MNIST dataset at different vocabulary sizes: 10 (top row), 20 (second row), 50 (third row), and 100 (bottom row). The first column belongs to agent A, while the second one belongs to agent B

2 Appendix 2 - Evaluation Metrics

- **Cohen's Kappa coefficient (Kappa)** [Cohen, 1960]: Measures inter-rater reliability and is used to assess how closely the observed categories match the categories expected by chance. The formula is:

$$\kappa = \frac{P_o - P_e}{1 - P_e} \tag{1}$$

  Where P_o is the observed proportion of agreement and P_e is the expected proportion of agreement if both raters assign categories randomly. Interpretation guidelines are:

  - $0.8 < \kappa \leq 1.0$: Almost perfect agreement
  - $0.6 < \kappa \leq 0.8$: Substantial agreement
  - $0.4 < \kappa \leq 0.6$: Moderate agreement
  - $0.0 < \kappa \leq 0.4$: Slight to fair agreement
  - $\kappa \leq 0.0$: No agreement

- **Adjusted Rand Index (ARI)** [Hubert and Arabie, 1985]: Compares two clusterings by considering all pairs of samples and counting pairs that are clustered in the same or different clusters in the predicted and true clusterings. It's robust to label switching effects in clustering. The formula is:

$$ARI = \frac{\sum_{ij} \binom{n_{ij}}{2} - \left[\sum_i \binom{a_i}{2} \sum_j \binom{b_j}{2}\right] / \binom{n}{2}}{\frac{1}{2}\left[\sum_i \binom{a_i}{2} + \sum_j \binom{b_j}{2}\right] - \left[\sum_i \binom{a_i}{2} \sum_j \binom{b_j}{2}\right] / \binom{n}{2}} \tag{2}$$

  Where n_{ij} is the number of data points common between cluster i in the ground truth and cluster j in the predicted clustering. Values higher are better, with $ARI = 1$ indicating perfect agreement while $ARI = 0$ suggests a random clustering.

- **Davies Bouldin Score (DBS)** [Davies and Bouldin, 1979]: Evaluates the quality of cluster assignments by relating the average similarity measure of each cluster with its most similar cluster. Lower values indicate better clustering.

$$DBS = \frac{1}{n} \sum_{i=1}^{n} \max_{i \neq j} \left(\frac{S_i + S_j}{d(i,j)} \right) \tag{3}$$

  Where S_i is the average distance between each point of cluster i and the centroid of that cluster. $d(i,j)$ is the distance between cluster centroids. The DBS metric is minimized for optimal clustering.

- **Fréchet Inception Distance (FID)** [Heusel et al., 2017]: Measures the distance between real and generated image distributions, offering a more robust metric than direct pixel-wise comparisons. A lower FID score indicates that the two samples are more similar, suggesting better quality for generated images.

$$FID = \|\mu_1 - \mu_2\|^2 + \mathrm{Tr}(\Sigma_1 + \Sigma_2 - 2(\Sigma_1\Sigma_2)^{0.5}) \tag{4}$$

  Where μ_1 and μ_2 are the sample means of the real and generated images, respectively, and Σ_1 and Σ_2 are their covariances. Lower values are better, indicating that the generated images are more similar to the real images.

3 Appendix 3 - The network architecture

Figure 2 presents the VAE network architecture used in experiment 1, designed for two distinct modalities. The top VAE is configured for the MNIST dataset, while the VAE in the lower is designed for the SVHN dataset.

Figure 3 illustrates the VAE network structure implemented in experiment 2, incorporating three VAEs for three separate modalities. The first VAE is used for the image or visual modality, the second is configured for the auditory modality, and the third VAE is dedicated to the haptic modality.

The terms ”Conv,” ”ConvTrans,” ”Linear,” ”ReLU,” and ”Sigmoid” refer to the convolutional layers, transposed convolutional layers, fully connected layers, the Rectified Linear Unit function, and the Sigmoid activation function, respectively. The notation “a*b*c” represents the data dimensions in terms of “channels*width*height.”

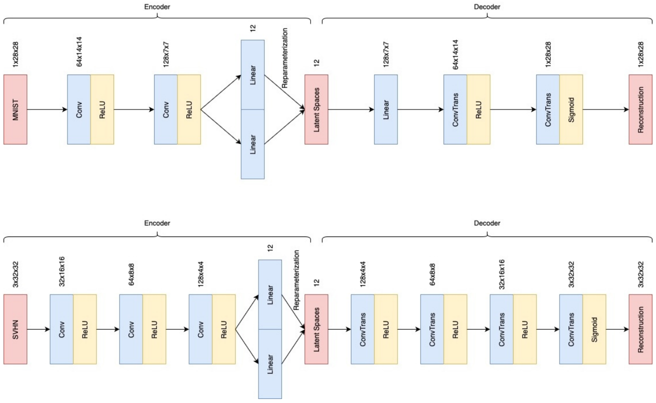

Figure 2: The network architecture of VAE used in experiment 1. The first is for MNIST, while the second is for SVHN.

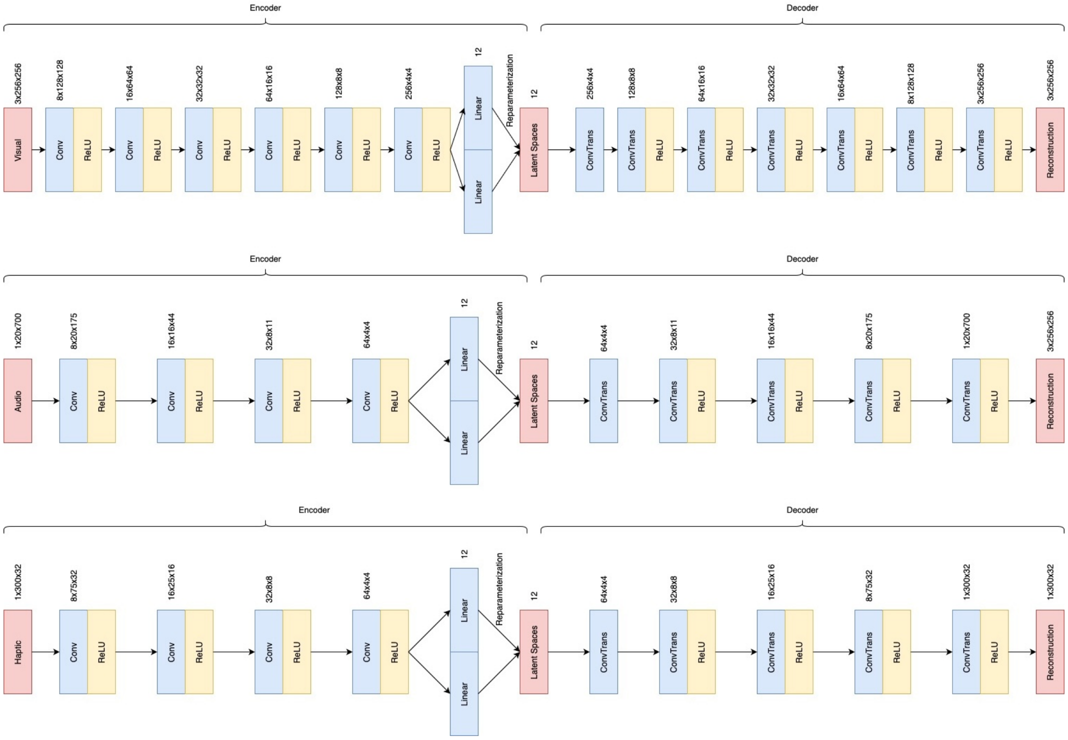

Figure 3: The network architecture of VAE used in experiment 2. The first is for vision modality, the second is for audio modality, and the last one is for haptic modality.

4 Appendix 4 - The visualizations from experiment 1

This appendix presents the visualizations from experiment 1: reconstructed data are displayed in Figures 4, 5, and 6, while the t-SNE visualization of latent spaces is shown in Figure 7.

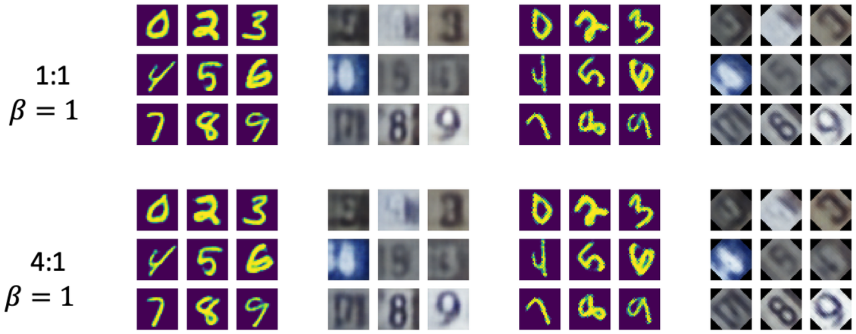

Figure 4: The reconstructed data of Inter-GMM+weighted-MoE-MVAE in experiment 1.

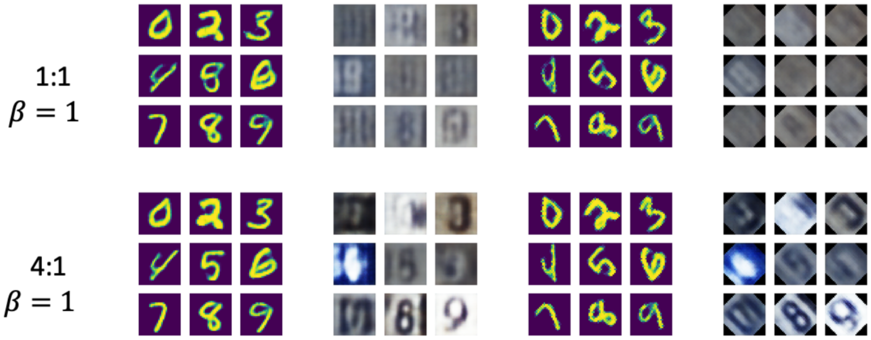

Figure 5: The reconstructed data of Inter-GMM+weighted-PoE-MVAE in experiment 1.

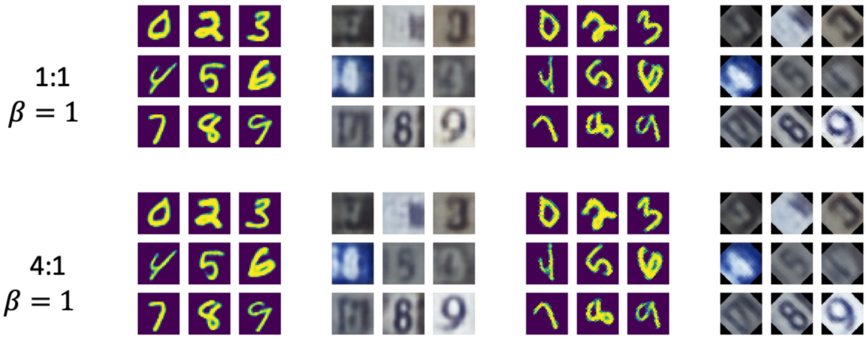

Figure 6: The reconstructed data of Inter-GMM+weighted-MoPoE-MVAE in experiment 1.

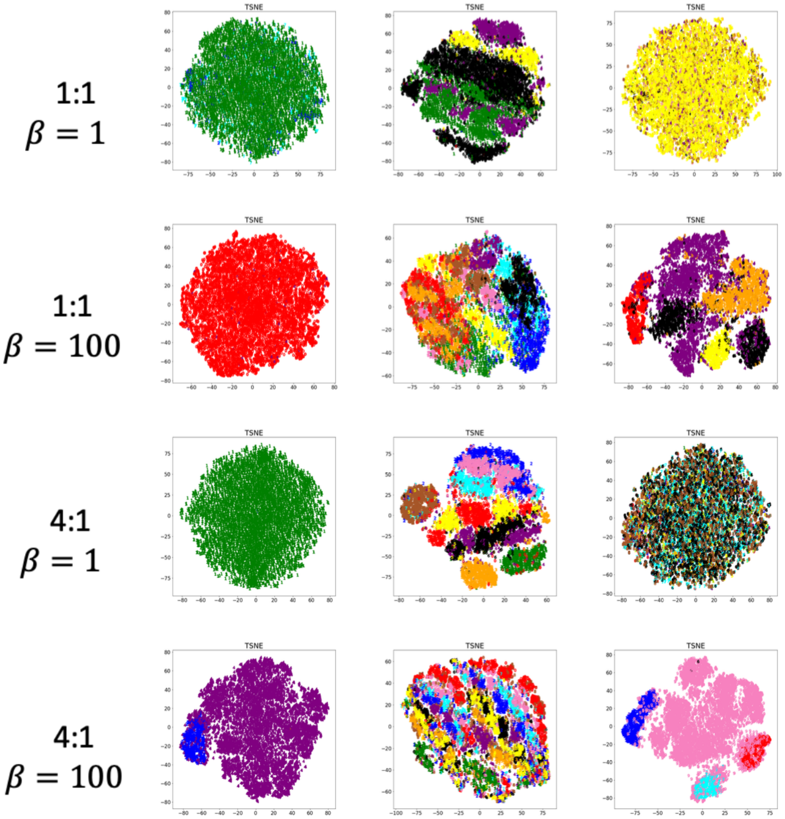

Figure 7: The t-SNE visualization of latent spaces of Inter-GMM+weighted-β-MVAE with MoE on the left column, PoE in the middle column, and MoPoE on the right column for experiment 1, illustrating the clustering of data across ten classes according to 10 digits.

References

[Cohen, 1960] Cohen, J. (1960). A coefficient of agreement for nominal scales. *Educational and Psychological Measurement*, 20(1):37–46.

[Davies and Bouldin, 1979] Davies, D. and Bouldin, D. (1979). A cluster separation measure. *IEEE Transactions on Pattern Analysis and Machine Intelligence*, 2:224–227.

[Heusel et al., 2017] Heusel, M., Ramsauer, H., Unterthiner, T., Nessler, B., and Hochreiter, S. (2017). Gans trained by a two time-scale update rule converge to a local nash equilibrium. *Advances in Neural Information Processing Systems*, pages 6626–6637.

[Hubert and Arabie, 1985] Hubert, L. and Arabie, P. (1985). Comparing partitions. *Journal of Classification*, 2(1):193–218.

[Taniguchi et al., 2023] Taniguchi, T., Yoshida, Y., Matsui, Y., Hoang, N. L., Taniguchi, A., and Hagiwara, Y. (2023). Emergent communication through metropolis-hastings naming game with deep generative models. *Advanced Robotics*, 37(19):1266–1282.
